# Population Structure and Spatial Connectivity Inferred from Spatial Phenotypic Differentiation of the Bluefish *Pomatomus saltatrix* (Teleostei: Pomatomidae) Along the Southeastern–Southern Brazilian Coast Using Landmark-Based Truss Network Morphometrics

**DOI:** 10.3390/biology15201883

**Published:** 2026-10-19

**Authors:** Bianca M. Neves, Felippe A. Daros, Rafael Schroeder, Rafael G. Kulzer, Marcus R. Costa, Rodolfo M. Silva, Alberto T. Correia

**Affiliations:** 1 Faculdade de Ciência e Tecnologia (FCT), Universidade Fernando Pessoa (UFP), Praça 9 de Abril 349, 4249-004 Porto, Portugal; 2 Centro Interdisciplinar de Investigação Marinha e Ambiental (CIIMAR/CIMAR), Terminal de Cruzeiros do Porto de Leixões, Avenida General Norton de Matos S/N, 4450-208 Matosinhos, Portugal; 3 Universidade Estadual Paulista (UNESP), Av. Nelson Brihi Badur 430, Registro 11900-000, São Paulo, Brazil; 4 Universidade do Vale do Itajaí (UNIVALI), Rua Uruguai 458, Itajaí 88302-901, Santa Catarina, Brazil; 5 Instituto de Ciências Biomédicas Abel Salazar (ICBAS), Universidade do Porto (UP), Rua Jorge Viterbo Ferreira 228, 4050-313 Porto, Portugal; 6 Universidade Federal Fluminense (UFF), Rua Professor Marcos Waldemar de Freitas Reis S/N, Campus do Gragoatá, Niterói 24210-201, Rio de Janeiro, Brazil

**Keywords:** fish populations, southwest Atlantic, natural tags, phenotypic differentiation

## Abstract

Understanding the population structure of highly migratory marine species is essential for effective fisheries management and conservation. The bluefish, *Pomatomus saltatrix*, is a widely distributed pelagic fish of great ecological and economic importance in the Southwest Atlantic; however, its population structure in this region remains poorly understood. This study investigated the population structure of *P. saltatrix* along the southeastern and southern coasts of Brazil using a landmark-based truss network morphometrics approach. A total of 109 adult individuals were sampled at three sites along the Brazilian coast—Rio de Janeiro, São Paulo and Santa Catarina—between August and October 2024. Body morphology was assessed using a truss network system composed of 25 morphometric transformed distances derived from 12 landmarks. One-Way ANOVA or Kruskal–Wallis tests revealed significant differences among sampling sites for 18 of the 25 transformed morphometric distances. PERMANOVA revealed significant overall differences in body morphology among the three sampling sites, with all pairwise comparisons being significant. Moreover, the FDA showed a clear spatial structure, despite partial overlapping between the three sampling-site groups. Overall jackknife reclassification success was 87.2%, with the highest accuracy achieved for Rio de Janeiro, followed by São Paulo and Santa Catarina. Despite the high reclassification accuracy, the observed morphometric differences may be compatible with a shared stock exhibiting site-specific phenotypic variation, although stock boundaries cannot be established from morphometric evidence alone. Overall, these findings demonstrate that landmark-based truss network morphometrics is an effective tool for detecting spatial phenotypic structuring in *P. saltatrix*, providing valuable information to support future studies and fisheries management in the Southwestern Atlantic.

## Simple Summary

The bluefish is an important fish species found along the Brazilian coast, valued both ecologically and economically. However, scientists did not fully understand how its populations are organized in the southwestern Atlantic Ocean. This study aimed to investigate whether bluefish from different parts of the Brazilian coast—Rio de Janeiro, São Paulo, and Santa Catarina—belong to the same population or form distinct groups. Researchers examined the body shapes of 109 adult fish collected between August and October 2024 using a method that measures specific physical landmarks on each fish. The results showed that fish from different sampling sites had noticeable differences in body shape, with a classification accuracy of 87.2%. However, these differences do not necessarily indicate separate populations. The findings may be compatible with a shared population showing differences in body shape among sampling sites, although further studies are needed to clarify population boundaries. This information is important because it helps fisheries managers make better decisions about how to fish sustainably and protect this species. Understanding population structure ensures that conservation efforts target the right groups, helping to maintain healthy fish populations and supporting the livelihoods of communities that depend on fishing.

## 1. Introduction

The bluefish, *Pomatomus saltatrix* (Linnaeus, 1766), is the only extant representative of the family Pomatomidae and is a highly migratory pelagic fish with a circumglobal distribution throughout temperate and subtropical waters of the Atlantic, Indian, and western Pacific oceans, being absent only from the eastern Pacific [1,2,3]. The species inhabits coastal and continental shelf habitats, where it occupies a high trophic level and plays an important ecological role in regulating coastal marine food webs through intense predatory activity [2,4,5]. Beyond its ecological importance, *P. saltatrix* supports valuable commercial and recreational fisheries throughout much of its geographic distribution [2,6]. In the Southwestern Atlantic, it represents one of the most economically important coastal pelagic fishery resources, supporting both artisanal and industrial fisheries and contributing substantially to regional fishery production [6,7,8]. Consequently, accurately identifying biologically meaningful population units is fundamental for the sustainable assessment, management, and conservation of this widely exploited resource, providing the scientific basis for defining appropriate management units and supporting effective fisheries management [9,10,11].

Understanding population structure in *P. saltatrix* is particularly challenging because its life history is closely linked to seasonal oceanographic variability, especially changes in sea surface temperature, which regulate migration patterns, spawning period, recruitment dynamics, and juvenile survival throughout its distribution [2,12,13]. These environmental conditions drive extensive seasonal migrations between feeding and spawning grounds while also promoting extended reproductive seasons and regional variation in spawning timing according to local oceanographic conditions [8,13,14]. During early ontogeny, juveniles use both estuarine and shallow coastal waters as nursery areas, although the relative importance of each habitat varies among regions and cohorts [15,16,17]. The use of multiple nursery habitats is expected to facilitate dispersal and increase the potential for population connectivity across broad geographic scales [16,18]. Although these biological characteristics suggest extensive population connectivity, increasing evidence demonstrates that high dispersal potential alone does not necessarily prevent the development of spatial population structure in marine fishes, particularly where environmental heterogeneity and local adaptation act simultaneously [18,19].

Across its broad geographic range, *P. saltatrix* inhabits coastal ecosystems characterized by pronounced environmental heterogeneity, including variations in temperature, primary productivity, hydrodynamic regimes, prey availability, and oceanographic conditions [2,3,4]. These environmental gradients influence habitat use, feeding ecology, growth rates, body condition, and energy allocation throughout different life stages, contributing to considerable ecological plasticity [4,5,20]. Ontogenetic shifts in feeding behavior occur early in development, with juveniles feeding primarily on zooplankton before progressively transitioning to a predominantly piscivorous diet as they grow [5,20]. Furthermore, the species exhibits considerable trophic flexibility by adjusting its feeding strategy according to local prey availability and environmental conditions through shifts in habitat use and expansion of its trophic niche [4,17]. Because body morphology integrates both inherited genetic information and environmentally induced developmental responses accumulated throughout an individual’s life, environmental variation affecting growth, body condition, and resource allocation may promote phenotypic divergence even among populations connected by substantial gene flow [21,22,23]. Consequently, environmentally driven morphological variation provides valuable insights into population structure and represents an important complement to genetic approaches for investigating spatial differentiation in highly migratory marine fishes [10,11,19].

Consistent with the influence of environmental variability on phenotypic divergence, molecular studies have revealed that the population structure of *P. saltatrix* is more complex than previously recognized. Phylogeographic analyses based on mitochondrial DNA have demonstrated that historical climatic fluctuations and oceanographic barriers have promoted marked genetic differentiation among populations from the North Atlantic and Mediterranean Sea, despite the species’ high dispersal capacity [24,25]. Building upon these findings, DNA barcoding analyses revealed five highly divergent Molecular Operational Taxonomic Units (MOTUs) across the species’ global distribution, suggesting the existence of cryptic evolutionary lineages and highlighting priority populations for conservation [26]. Furthermore, complete mitochondrial genome analyses revealed two Evolutionarily Significant Units (ESUs) along the Southwestern Atlantic, separated by a transition zone near southeastern Brazil, providing strong evidence that the Brazilian coast harbors biologically meaningful population subdivisions despite the species’ high migratory capacity [26,27].

Recognizing biologically meaningful population units is fundamental for the sustainable management of exploited marine fishes because exploitation strategies based on incorrectly defined stocks may compromise recruitment, reduce population resilience, and ultimately increase the risk of local population declines [9,10,11]. In the Southwestern Atlantic, *P. saltatrix* supports artisanal and industrial fisheries and is intensively exploited, representing one of the most important coastal pelagic fishery resources in southern and southeastern Brazil exploited by purse-seine and gillnet fleets [6,7,8]. Although artisanal fishers have reported regional declines in abundance [28], official statistics indicate that recorded nationwide Brazilian catches of *P. saltatrix* increased from 5780 t in 2023 to 11,300 t in 2024 [29]. However, these national catch figures are not directly comparable with historical, region-specific observations of declining abundance. Moreover, changes in reported catches do not necessarily reflect changes in stock abundance, particularly given potential differences in fishing effort and limitations in fisheries monitoring. Reproductive studies conducted along the Brazilian coast estimated the total length at which 50% of individuals reach sexual maturity (L50) to be 35.5 cm for females and 38.3 cm for males, indicating that the capture of immature individuals may compromise reproductive output and long-term stock sustainability [8]. These findings reinforce the importance of accurately defining population units to support fisheries management strategies capable of preserving recruitment, maintaining population resilience, and ensuring the long-term sustainability of this valuable fishery resource [9,10,11].

Accordingly, phenotypic approaches have become increasingly valuable for investigating stock structure because biologically meaningful population units cannot always be fully resolved using genetic markers alone [10,11,30]. Body morphology reflects the combined influence of genetic inheritance and environmental conditions experienced throughout the life cycle, making morphometric analyses effective tools for detecting spatial population differentiation among fish populations [10,21,22]. Among these approaches, landmark-based truss network analysis provides a comprehensive description of body shape and has successfully identified subtle population differentiation in numerous marine fishes with high dispersal potential [31,32,33]. Its high repeatability, low cost, and ability to integrate environmentally and genetically influenced variation have made it an effective tool for stock discrimination in both marine and freshwater fishes [11,34,35]. More recently, studies conducted along the Brazilian coast have demonstrated that body morphometry effectively detects geographic population differentiation and complements molecular approaches for investigating stock structure in marine fishes [36,37,38].

Despite substantial advances in understanding the ecology, genetics, and fisheries biology of *P. saltatrix* [8,27], important uncertainties remain regarding its population structure along the southeastern–southern Brazilian coast. This region supports one of the most important fisheries for the species in the Southwestern Atlantic, highlighting the importance of improving our understanding of stock structure [6,7,8]. Evaluating phenotypic divergence among individuals from distinct fishery grounds may therefore provide complementary evidence for understanding population structure, improving stock delineation, and supporting more effective fisheries management and conservation strategies. Therefore, the present study aimed to investigate the population structure of *P. saltatrix* from three sampling sites along the southeastern–southern Brazilian coast using a landmark-based truss network approach, evaluating spatial variation in body morphology and assessing whether phenotypic differentiation reflects population structuring within the Southwestern Atlantic.

## 2. Materials and Methods

### 2.1. Fish Sampling

**Figure 1:**
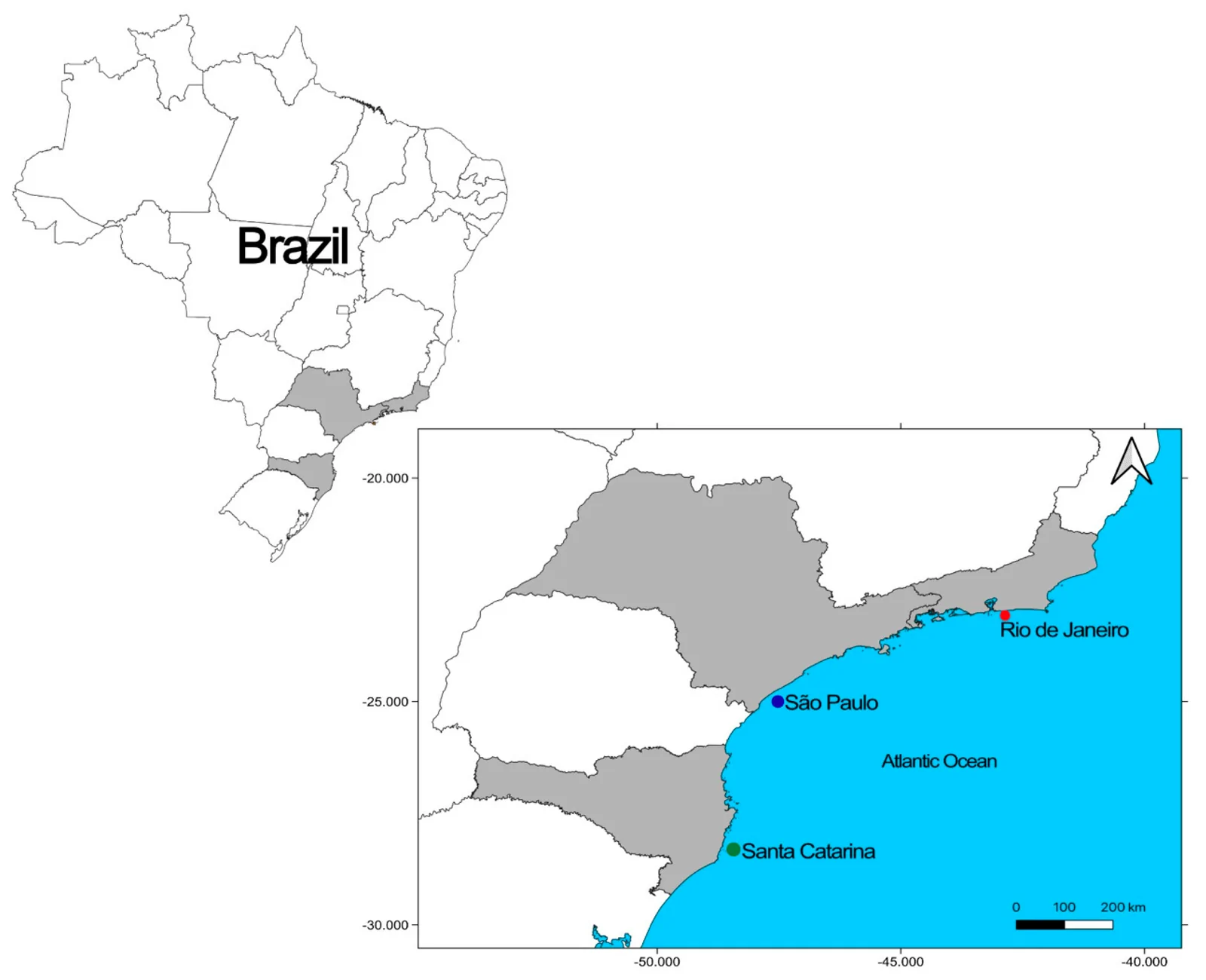
Map of southeastern and southern Brazil showing the three sampling sites [Rio de Janeiro (red dot), São Paulo (blue dot), and Santa Catarina (green dot)] where *Pomatomus saltatrix* specimens used in this study were collected between August and October 2024. The inset (upper left corner) indicates the location of the study area (grey colour) within Brazil.

Individuals of *P. saltatrix* were collected from three sampling sites along the southeastern–southern Brazilian coast: Niterói (Rio de Janeiro), Cananéia (São Paulo), and Laguna (Santa Catarina) between August and October 2024 (Figure 1). Most individuals (88.1%) had a total length (TL) equal to or greater than the overall estimated length at 50% sexual maturity (TL_50_ = 37.4 cm), with sex-specific estimates of 38.3 cm for males and 35.5 cm for females [8]. In addition, all specimens were collected within a three-month interval (August to October), thereby minimizing the potential effects of ontogenetic and short-term seasonal variation in body morphology. A total of 109 individuals were analyzed, with TL ranging from 32.0 to 48.0 cm (overall mean ± SE: 42.1 ± 0.3 cm), including 46 individuals from Rio de Janeiro, 35 from São Paulo, and 28 from Santa Catarina (Table 1). TL differed significantly among sampling sites (One-Way ANOVA, DF = 2,106, F = 9.420, *p* < 0.001). Tukey’s HSD test revealed that individuals from São Paulo were significantly smaller than those from Rio de Janeiro (*p* < 0.001) and Santa Catarina (*p* = 0.013), whereas no significant differences were detected between Rio de Janeiro and Santa Catarina (*p* = 0.630; Table 1). Specimens were obtained directly from local artisanal fishers at beach landing sites immediately after capture, ensuring that the landing site corresponded to the capture location. At some sampling locations, specimens were collected on multiple occasions throughout the sampling month, depending on fish availability and fishing activity. Three fishing gear types were used at all sampling sites (Rio de Janeiro, São Paulo, and Santa Catarina): gillnets, purse seines, and longlines. However, detailed technical specifications of the fishing gears, including mesh sizes and hook characteristics, were not available.

After capture, all specimens were placed on ice and transported to the laboratory for morphometric analyses. TL (0.1 cm) was measured for all individuals. The left lateral side of each fresh specimen was photographed using a standardized positioning protocol. Temporary pins were inserted to improve the visualization of anatomical landmarks, particularly at the insertions of the fins, ensuring accurate landmark placement.

**Figure 2:**
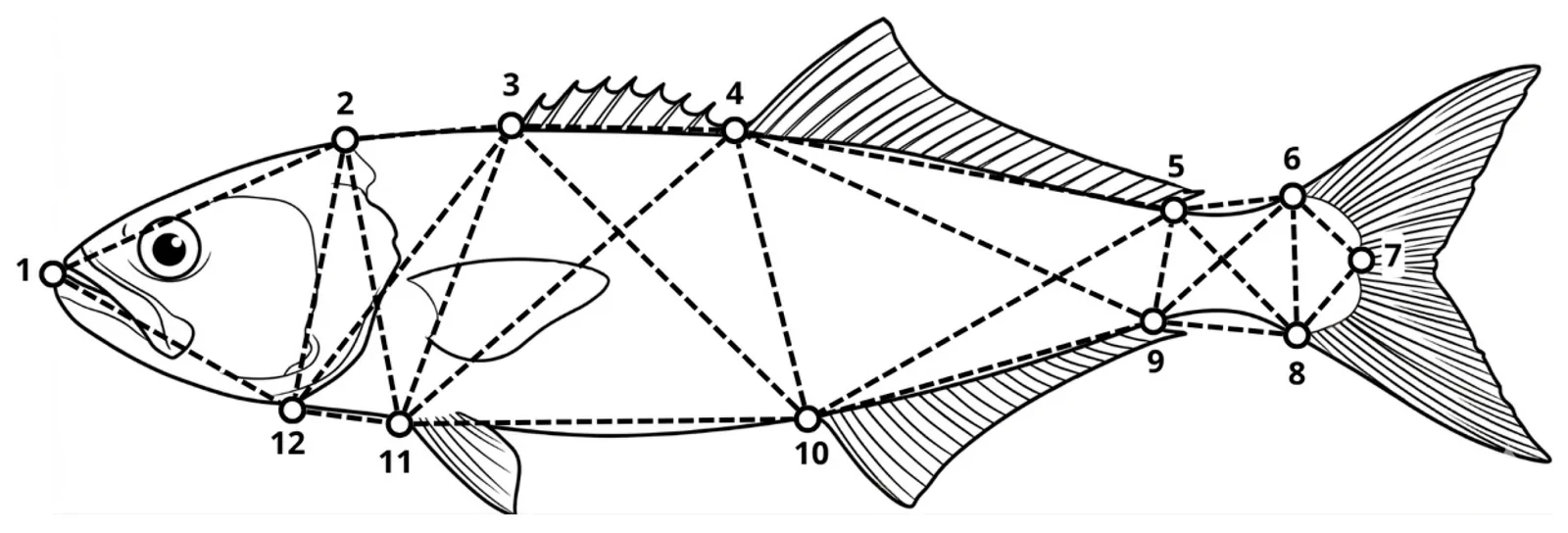
Location of the 12 landmarks (numbers) on the left side of the fish and the corresponding body morphometric distances (dashed lines). For more information, see also Table 2.

Prior to spatial comparisons, the potential effects of fish size on morphometric measurements were assessed using analysis of covariance (ANCOVA), with sampling site as a fixed factor and standard length (SL) as a covariate. A significant positive relationship between SL and each of the 25 morphometric distances (D) was detected (*p* < 0.05 in all cases). To remove the effects of allometric growth on body shape, all morphometric distances were size-corrected using the allometric transformation proposed by [39]: DT = 10ˆ[log(D) − β [log(SL) − log(SLmean)]], where *DT* is the size-adjusted morphometric distance, *D* is the original morphometric distance, *β* is the slope of the linear regression between each log-transformed morphometric distance (D) and the log-transformed standard length (SL), estimated separately for each of the 25 morphometric distances, *SL* is the standard length of each individual, and *SL* mean is the grand mean standard length of all individuals included in the analysis [39]. SL, instead of TL, measured as the distance between landmarks 1 and 7 (Figure 2), was used for the allometric correction of morphometric distances. All 109 specimens had complete measurements for the 25 truss distances, and no missing values were present in the morphometric dataset.

### 2.2. Truss Network Morphometrics

Body morphology of *P. saltatrix* was quantified using the original landmark-based Truss Network system based on anatomical landmarks [31,39,40], following standardized protocols [41,42,43]. This approach provides a practical, repeatable, and sensitive method for detecting subtle morphological variation among fish populations [43,44,45].

For morphometric analyses, each specimen was photographed from a fixed distance with the fins fully extended. A 10 mm scale was included in each image for calibration, and photographs were acquired using a high-resolution Sony IMX363 digital camera (12 MP CMOS sensor; image resolution: 4032 × 3024 pixels; Sony Corporation, Tokyo, Japan).

A total of 12 homologous anatomical landmarks (Figure 2; Table 2) were identified at easily recognizable anatomical locations, generating 25 morphometric distances describing overall body shape [46]. Anatomical landmarks were manually marked on the standardized digital images using TPSUtil version 1.76 and TPSDig2 version 2.31 [47]. The resulting Cartesian coordinates (X and Y) were used to calculate the Euclidean distances between the corresponding landmark pairs. These distances were subsequently converted into metric units using the photographic scale, yielding the 25 morphometric distances defining the truss network [35,41,42]. All anatomical landmarks were marked by a single observer, blinded to the sampling location of each specimen. The resulting morphometric distances were subsequently checked for consistency to identify potential landmark positioning or calculation errors.

Although specimens were not sexed, previous research on *P. saltatrix* using truss network morphometrics characters found no significant differences between males and females ([34]: MANOVA, Wilks’ λ = 0.456, *p* = 0.172), supporting the pooling of sexes in morphometric analyses.

### 2.3. Statistical Analyses

Morphometric distances were subjected to univariate and multivariate statistical analyses to evaluate morphological differences among the three sampling sites. Prior to the analyses, normality and homogeneity of variances were assessed using the Shapiro–Wilk and Brown–Forsythe tests, respectively [48]. Variables meeting the assumptions of normality and homoscedasticity were analyzed using One-Way Analysis of Variance (ANOVA), followed by Tukey’s multiple comparison test when significant differences were detected (*p* < 0.05). Variables that violated these assumptions were analyzed using the Kruskal–Wallis test, followed by Dunn’s multiple comparison test whenever significant differences were observed (*p* < 0.05).

Overall multivariate differences in body morphology among sampling sites were assessed using Permutational Multivariate Analysis of Variance (PERMANOVA) based on the altGower distance measure with 9999 permutations, with sampling site included as a fixed factor with three levels. Homogeneity of multivariate dispersion among sampling sites was evaluated using PERMDISP, based on distances to group centroids, to assess whether differences in within-group dispersion could influence the PERMANOVA results. When significant differences were detected (*p* < 0.05), pairwise PERMANOVA comparisons were subsequently performed to identify which sampling sites differed in body morphology, with *p*-values adjusted using the Bonferroni correction to account for multiple comparisons.

Morphometric distances were further analyzed using Flexible Discriminant Analysis (FDA) to assess the ability of body morphology to discriminate individuals among sampling sites [49,50,51]. FDA extends Linear Discriminant Analysis (LDA) by incorporating nonlinear transformations of predictor variables, such as splines, allowing greater flexibility in modelling complex relationships between morphometric variables and group membership [50,51]. The dataset was randomly divided into training (80%) and test (20%) subsets. Predictor variables were centered and scaled using the means and standard deviations estimated from the training set, and the same transformations were subsequently applied to the test set. Classification accuracy was calculated as the percentage of individuals correctly assigned to their original sampling locations. Additionally, a leave-one-out (jackknife) cross-validation procedure was applied to the full dataset to assess reclassification accuracy [52].

Univariate statistical analyses were performed using SYSTAT version 13.0 and SigmaPlot version 16.0, whereas PERMANOVA and FDA were performed in R (version 4.4.3). The level of statistical significance was set at α = 0.05.

## 3. Results

Most morphometric distances were significantly different among sampling sites, except for DT3, DT4, DT6, DT9, DT13, DT24, and DT25 (One-Way ANOVA or Kruskal–Wallis, *p* < 0.05). Pairwise comparisons performed using Tukey’s or Dunn’s post-hoc tests (*p* < 0.05) identified three morphometric distances (DT14, DT16, and DT19) that differed significantly among all sampling sites (Table 3). DT1 distinguished Rio de Janeiro from both São Paulo and Santa Catarina, whereas DT2, DT10, DT18, DT22, and DT23 distinguished São Paulo from the other sampling sites. In contrast, DT20 and DT21 differentiated Santa Catarina from Rio de Janeiro and São Paulo (Table 3).

PERMANOVA revealed significant differences in body morphology among sampling sites (Pseudo-F_2,106_ = 8.797, R^2^ = 0.142, *p* < 0.001; Table 4). Pairwise comparisons showed significant differences between all sites after adjustment for multiple comparisons (*p* ≤ 0.001). The greatest morphological differentiation was observed between São Paulo and Santa Catarina (R^2^ = 0.202), followed by Rio de Janeiro and São Paulo (R^2^ = 0.086), whereas the smallest difference was detected between Rio de Janeiro and Santa Catarina (R^2^ = 0.074) (Table 4). However, PERMDISP revealed significant heterogeneity in multivariate dispersion among locations (F_2,106_ = 11.44, *p* = 0.010), with significant differences between São Paulo and both Rio de Janeiro and Santa Catarina (*p* = 0.010), but not between Rio de Janeiro and Santa Catarina (*p* = 0.900). These findings suggest that differences in within-group dispersion may have contributed to the PERMANOVA results, particularly in comparisons involving São Paulo.

**Figure 3:**
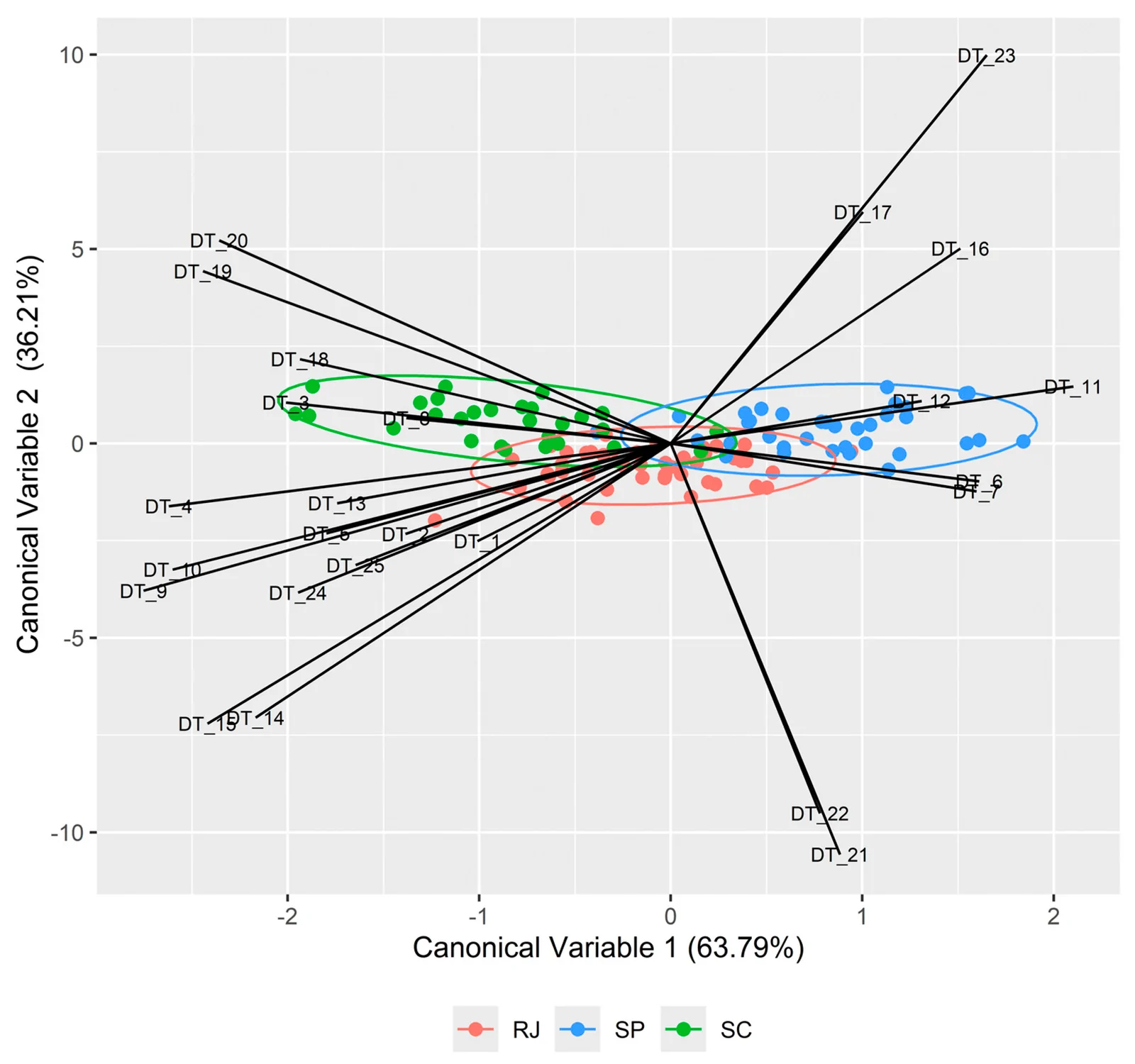
Flexible Discriminant Analysis (FDA) plot displaying spatial differences for truss network (Morphometric Transformed Distances, DT) from *Pomatomus saltatrix*. Ellipses represent 95% confidence intervals around the data, and each data point represents an individual fish. Abbreviations: Rio de Janeiro (RJ, red dots), São Paulo (SP, blue dots) and Santa Catarina (SC, green dots).

FDA revealed partial overlap among individuals from the three sampling sites (Figure 3). Despite this overlap, jackknife cross-validation achieved an overall reclassification accuracy of 87.2%**.** Reclassification accuracy was highest for Rio de Janeiro (89.1%) and São Paulo (88.6%), whereas Santa Catarina showed a comparatively lower, although still high, reclassification accuracy (82.1%) (Table 5).

## 4. Discussion

The present study revealed significant spatial variation in the body shape of *P. saltatrix* along the southeastern–southern Brazilian coast, with clear morphometric differentiation among specimens from Rio de Janeiro, São Paulo, and Santa Catarina. The landmark-based truss network approach proved effective in detecting subtle phenotypic differences, highlighting the usefulness of quantitative body-shape analyses for investigating population structure in marine fishes [35,36,44]. The observed morphometric patterns are most consistently explained by the combined influence of environmental heterogeneity, phenotypic plasticity, and historical evolutionary processes rather than by complete reproductive isolation. These findings reveal spatial phenotypic differentiation in *P. saltatrix* along the southeastern–southern Brazilian coast, which may coexist with the seasonal connectivity previously suggested by ecological studies [3,4,26]. However, the observed morphometric patterns do not independently establish demographic connectivity or the boundaries of fisheries management units.

Although sampling was conducted at different locations between August and October 2024, most specimens had total lengths equal to or greater than the estimated length at 50% sexual maturity (L50 = 37.4 cm TL) [8]. Furthermore, published age–growth relationships for *P. saltatrix* in southern Brazil indicate that the mean total lengths observed in the present study (approximately 40–43 cm) are consistent with individuals around 2–3 years old [53]. Although individual ages were not determined, these observations suggest that the specimens had undergone substantial ontogenetic development and that their body shape reflects growth and developmental processes accumulated over several years. Consequently, the relatively short interval between sampling campaigns is unlikely, by itself, to explain the observed morphometric differentiation. Nevertheless, because sampling location and month were confounded, their independent effects could not be formally assessed. Moreover, sampling a single locality per region limits the extrapolation of the observed patterns beyond the sampled localities.

The relatively modest proportion of morphometric variation explained by sampling location in PERMANOVA (R^2^ = 0.142, corresponding to 14.2% of the total multivariate variation) contrasts with the high classification accuracy achieved by FDA. However, these findings are not necessarily contradictory, as PERMANOVA quantifies the contribution of sampling location to overall multivariate variation, whereas FDA identifies combinations of morphometric variables that maximize discrimination among groups. Furthermore, the significant differences in multivariate dispersion detected by PERMDISP (*p* = 0.010) suggest that the PERMANOVA results should be interpreted cautiously, as they may partly reflect differences in within-site variability rather than differences in group centroids alone. Nevertheless, high classification accuracy should not be interpreted as direct evidence of distinct population stocks, particularly given the relatively modest proportion of multivariate variation explained by sampling location.

Significant phenotypic differences in body shape were detected among the three sampling sites, indicating spatial phenotypic differentiation in *P. saltatrix* along the southeastern–southern Brazilian coast. As a phenotypic approach, body morphometrics has been widely applied to investigate population structure because body shape integrates both genetic and environmental influences acting throughout individual development [35,36,42]. However, body morphology also exhibits considerable phenotypic plasticity, and morphometric differences alone should not be interpreted as unequivocal evidence of reproductive isolation, since they may reflect adaptive responses to regional environmental conditions [10,32,54]. Therefore, the phenotypic differentiation observed in the present study indicates that *P. saltatrix* is not phenotypically homogeneous along the southeastern–southern Brazilian coast. However, these differences are more consistent with spatial structuring within a single stock than with complete population segregation, reinforcing the need to integrate phenotypic, genotypic, and ecological evidence when inferring population boundaries [11,27,54].

Although significant phenotypic differences were detected among the sampling sites, previous ecological evidence suggests that *Pomatomus saltatrix* maintains extensive seasonal movements and substantial connectivity throughout the Southwestern Atlantic [6,8,53]. Seasonal latitudinal migration has long been considered the primary mechanism maintaining population connectivity in this region, based on consistent evidence from fishery records, age composition, and movement patterns [6,7,53]. This evidence supports the hypothesis that individuals distributed between Rio de Janeiro (23° S) and Buenos Aires Province (38° S) belong to a single migratory stock that performs seasonal movements along the continental shelf [53]. This hypothesis has subsequently been reinforced by commercial fisheries, which consistently show that catches in southern Brazil are dominated by juvenile and young adult individuals during the winter migration period, as well as by ichthyoplankton surveys documenting a progressive northward distribution of *P. saltatrix* larvae associated with these seasonal movements [6,7,8,15]. Collectively, these ecological observations supported the long-standing hypothesis that *P. saltatrix* comprises a single, highly connected stock throughout much of the Southwestern Atlantic [6,8,53].

The long-standing hypothesis of a single, highly connected stock has been progressively re-evaluated as molecular markers revealed historical patterns of population divergence that could not be detected using phenotypic approaches alone [26,55,56]. Mitochondrial DNA analyses revealed five MOTUs distributed across the Atlantic Ocean, providing evidence of pronounced historical genetic structuring among regional lineages, as reflected by high FST values [26]. Samples from Brazil and Venezuela were assigned to MOTU V, which exhibited relatively low haplotype richness despite the high overall genetic diversity observed for the species, a pattern interpreted as evidence of historical demographic bottlenecks or prolonged periods of isolation [26,55,57]. Rather than contradicting the ecological evidence of seasonal migration, these findings indicate that historical genetic divergence can coexist with contemporary ecological connectivity [11,26,58]. Consequently, the phenotypic differentiation detected in the present study should be interpreted as spatial structuring within a connected population rather than evidence of fully discrete populations. This interpretation is consistent with the coexistence of contemporary ecological connectivity and historical genetic divergence suggested by previous molecular studies [11,26,56].

Recent genomic evidence further supports the coexistence of historical divergence and contemporary connectivity in *P. saltatrix* throughout the Southwestern Atlantic [27]. An integrated genomic approach identified two ESUs along the South American coast, with complete mitochondrial genome analyses providing independent support for this evolutionary subdivision [27]. The persistence of gene flow despite historical divergence suggests that evolutionary differentiation has not resulted in complete demographic isolation, a pattern consistent with expectations for highly migratory marine fishes [27,59,60]. These findings reinforce the view that historical genetic divergence and contemporary connectivity are not mutually exclusive processes in highly migratory marine fishes [11,27,60]. Consequently, the phenotypic differentiation detected among Rio de Janeiro, São Paulo, and Santa Catarina may reflect a combination of genetic, developmental, and environmental influences, although their relative contributions cannot be determined from the present morphometric data [11,27,60].

The two ESUs described for the Southwestern Atlantic display a predominantly parapatric distribution, with a seasonally variable zone of overlap near 23°40′ S, which coincides with the coastal region of Rio de Janeiro [27]. Even within this overlap zone, genetic differentiation between the two ESUs remains high, indicating that ecological barriers and local adaptation help maintain historical genetic divergence despite seasonal contact [27]. This evolutionary scenario provides a plausible biological context for interpreting the morphometric patterns observed in the present study, as the Rio de Janeiro sampling site is located within the transition zone between both ESUs [11,27,60]. However, pairwise PERMANOVA revealed the greatest morphological differentiation between São Paulo and Santa Catarina, whereas comparisons involving Rio de Janeiro showed smaller effect sizes. Furthermore, PERMDISP revealed significant differences in multivariate dispersion involving São Paulo, but not between Rio de Janeiro and Santa Catarina, providing no clear support for increased morphological variability associated with the proposed ESU contact zone. Therefore, the potential influence of historically divergent evolutionary lineages on body shape variation in Rio de Janeiro remains a hypothesis requiring further investigation. Previous studies suggest that body morphology in *P. saltatrix* may be influenced by individual growth history and environmental conditions, including temperature, food availability, oceanographic conditions, and habitat characteristics. Accordingly, environmental plasticity and historical evolutionary processes represent potential, non-mutually exclusive explanations for the phenotypic variation observed in the present study [3,11,61].

Oceanographic differences along the southeastern–southern Brazilian coast provide a relevant ecological context for discussing potential mechanisms underlying the body-shape differentiation observed among the sampling sites. In particular, the Cabo Frio upwelling system increases nutrient availability and primary productivity, modifying prey availability and trophic conditions, whereas the Santa Catarina coast is characterized by cooler, more homogeneous oceanographic conditions [3,62,63]. São Paulo occupies an intermediate oceanographic setting between these contrasting environments, reflecting transitional oceanographic conditions that reinforce the existence of a gradual environmental gradient across the study area [62,63,64]. These environmental contrasts provide a plausible ecological context for interpreting regional differences in body shape, although the present study did not directly assess environmental influences or distinguish environmentally mediated phenotypic plasticity from genetic differentiation [10,11,54]. Similar associations between morphometric differentiation and environmental heterogeneity have been reported for other marine fishes along the Brazilian coast, including *Isopisthus parvipinnis*, *Sardinella brasiliensis*, and *Mugil liza* [36,37,38].

The significant morphometric differences observed in the present study were distributed across multiple anatomical regions rather than being restricted to a single body structure, indicating that phenotypic divergence in *P. saltatrix* involves coordinated modifications of overall body shape. Significant variation was detected in head-related measurements, body depth, the relative position of the dorsal, anal and pelvic fins, and particularly in several dimensions of the caudal peduncle, suggesting that both anterior and posterior body regions contribute to spatial phenotypic differentiation. Because these anatomical regions are associated with feeding performance, swimming efficiency, and locomotor stability, the observed variation may have functional relevance. However, its relationship with environmental conditions or local selective pressures remains hypothetical, as these factors were not directly investigated in the present study [11,65,66]. Similar patterns have been described for other marine fishes inhabiting environmentally heterogeneous coastal systems, where morphometric divergence has been interpreted as the result of environmentally mediated phenotypic plasticity acting together with local selective pressures [32,35,36].

The results obtained in the present study demonstrate that landmark-based truss network morphometrics is sufficiently sensitive to detect subtle spatial phenotypic variation in a highly migratory species such as *P. saltatrix*. The approach successfully identified significant body-shape differences among the sampled locations, providing evidence of spatial phenotypic differentiation without establishing the underlying environmental or genetic mechanisms. These findings reinforce the usefulness of body morphometrics as a complementary tool for investigating population structure, particularly when interpreted together with independent ecological and molecular evidence [35,36,44].

Previous studies using truss network morphometrics have reported spatial phenotypic differentiation in *P. saltatrix* across the Black Sea, Sea of Marmara, Aegean Sea, and northeastern Mediterranean, highlighting the applicability of body morphology to investigate geographic variation in this widely distributed species [34,67]. More recently, otolith shape variation in *P. saltatrix* from the same three Brazilian coastal sites was investigated in a separate study, using Elliptic Fourier and Wavelet Transform Descriptors [68]. Although statistically significant spatial differences were detected, the proportion of variation explained by sampling location was low, indicating weak phenotypic differentiation based on otolith shape. Considered together, these findings demonstrate that different morphological structures can reveal spatial phenotypic heterogeneity in *P. saltatrix*, although differences in the magnitude of differentiation and discriminatory performance should be interpreted in light of the distinct biological characteristics and analytical approaches involved. Importantly, neither body nor otolith morphology alone can establish demographic connectivity or delimit discrete population units.

Overall, the present findings demonstrate significant body-shape differentiation among the sampled locations along the southeastern–southern Brazilian coast. Environmental heterogeneity, phenotypic plasticity, and evolutionary history represent plausible, potentially interacting mechanisms underlying patterns, although their individual contributions remain unresolved [3,11,27]. Nevertheless, the inclusion of a single sampling location per region and slightly unequal sample sizes should be considered when interpreting the observed morphometric differences, which may partly reflect local rather than broader regional patterns. Although truss network morphometrics alone cannot resolve population boundaries, it proved sufficiently sensitive to detect biologically meaningful spatial phenotypic variation that is consistent with recent ecological and genomic evidence [11,32,36]. Therefore, a holistic approach by integrating phenotypic, genotypic and ecological tools provides a more robust framework for understanding population structure and should be encouraged in future studies aiming to improve stock discrimination and fisheries management of this highly migratory species [11,27,54].

## 5. Conclusions

The present study demonstrated significant spatial variation in the body shape of *P. saltatrix* along the southeastern–southern Brazilian coast. The landmark-based truss network approach proved effective in detecting subtle phenotypic differences among specimens from Rio de Janeiro, São Paulo, and Santa Catarina, highlighting the usefulness of truss network morphometrics for investigating population structure in highly migratory marine fishes. Despite the high reclassification accuracy achieved by the morphometric analyses, the observed differences do not necessarily indicate the existence of distinct population stocks and may be compatible with a shared stock exhibiting site-specific phenotypic variation. However, stock boundaries cannot be reliably established from morphometric evidence alone. The observed morphological variation may reflect the combined influence of environmental heterogeneity, phenotypic plasticity, and historical evolutionary processes, although their relative contributions remain unresolved. The present findings provide valuable baseline information for stock discrimination and may contribute to the development of more effective management and conservation strategies for this economically important species.

## Figures and Tables

**Table 1:** Sampling sites, collection date, sample size (N) and total length (TL, cm) (mean values *±* standard error and range) of *Pomatomus saltatrix* used for body morphometry.

State	Site	Date	N	TL	
Rio de Janeiro	Niterói-Macaé	18 October 2024	46	43.0 ± 0.6	32.0–48.0
São Paulo	Cananéia	14 August 2024	35	40.8 ± 0.4	35.9–45.5
Santa Catarina	Laguna	22/25 September 2024	28	42.3 ± 0.4	38.0–46.0

**Table 2:** Anatomic landmarks defined along the body of *Pomatomus saltatrix* and morphometric distances used for the truss network analysis. For more details, please see also Figure 2.

**Body Landmarks**		
**Number**	**Location**	
1	Anterior tip of the mouth	
2	Posterior margin of the neurocranium	
3	Anterior insertion of the first dorsal fin	
4	Anterior insertion of the second dorsal fin	
5	Posterior insertion of the second dorsal fin	
6	Dorsal insertion of the caudal fin	
7	Posterior margin of the caudal peduncle	
8	Ventral insertion of the caudal fin	
9	Posterior insertion of the anal fin	
10	Anterior insertion of the anal fin	
11	Anterior insertion of the pelvic fin	
12	Ventral insertion of the operculum	
**Morphometric Distances**		
**Distances**	**Landmarks**	**Description**
D1	1 to 2	Head length
D2	1 to 12	Distance from snout to the ventral insertion of the operculum
D3	2 to 12	Head depth
D4	11 to 12	Distance from the pelvic fin to the ventral insertion of the operculum
D5	2 to 3	Distance from the posterior margin of the neurocranium to the anterior insertion of the first dorsal fin
D6	3 to 12	Distance from the anterior insertion of the first dorsal fin to the ventral insertion of the operculum
D7	2 to 11	Distance from the posterior margin of the neurocranium to the pelvic fin
D8	3 to 11	Anterior body depth
D9	3 to 4	First dorsal fin base length
D10	10 to 11	Distance from the anterior tip of the anal fin to the pelvic fin
D11	3 to 10	Distance from the anterior tip of the first dorsal fin to the anterior tip of the anal fin
D12	4 to 11	Distance from the anterior tip of the second dorsal fin to the pelvic fin
D13	4 to 10	Posterior body depth
D14	9 to 10	Anal fin base length
D15	4 to 5	Second dorsal fin base length
D16	5 to 10	Distance from the posterior tip of the second dorsal fin to the anterior tip of the anal fin
D17	4 to 9	Distance from the anterior tip of the second dorsal fin to the posterior tip of the anal fin
D18	5 to 9	Anterior depth of the caudal peduncle
D19	5 to 6	Dorsal length of the caudal peduncle
D20	8 to 9	Ventral length of the caudal peduncle
D21	5 to 8	Anterior diagonal of the caudal peduncle
D22	6 to 9	Posterior diagonal of the caudal peduncle
D23	6 to 8	Posterior depth of the caudal peduncle
D24	7 to 8	Distance from the posterior margin of the caudal peduncle to the ventral insertion of the caudal fin
D25	6 to 7	Distance from the dorsal insertion of the caudal fin to the posterior margin of the caudal peduncle

**Table 3:** Results of univariate statistics for transformed body morphometric distances (DT) calculated for *Pomatomus saltatrix* collected in Rio de Janeiro (RJ), São Paulo (SP) and Santa Catarina (SC). Values are presented as mean *±* standard error. For each row, variables with different superscript letters between sites indicate statistically significant differences. Depending on whether the assumptions of normality and homoscedasticity were met, differences among groups were assessed using either a One-Way ANOVA followed by Tukey’s post-hoc pairwise test (*p* < 0.05) or a Kruskal–Wallis test followed by Dunn’s post-hoc pairwise test (*p* < 0.05). For more details, see M&M.

	RJ	SP	SC
**Transformed Distances**			
DT1	7.5 ± 0.1 ^a^	8.0 ± 0.1 ^b^	8.0 ± 0.1 ^b^
DT2	7.0 ± 0.1 ^a^	7.2 ± 0.1 ^b^	6.7 ± 0.1 ^a^
DT3	7.1 ± 0.1 ^a^	7.2 ± 0.1 ^a^	7.1 ± 0.0 ^a^
DT4	4.2 ± 0.1 ^a^	4.3 ± 0.1 ^a^	4.5 ± 0.1 ^a^
DT5	6.4 ± 0.2 ^a^	6.1 ± 0.1 ^ab^	5.6 ± 0.2 ^b^
DT6	10.3 ± 0.1 ^a^	10.4 ± 0.1 ^a^	10.2 ± 0.2 ^a^
DT7	8.8 ± 0.1 ^a^	8.8 ± 0.0 ^a^	8.5 ± 0.1 ^b^
DT8	9.0 ± 0.1 ^ab^	9.1 ± 0.1 ^a^	8.8 ± 0.1 ^b^
DT9	8.0 ± 0.1 ^a^	7.6 ± 0.1 ^a^	7.7 ± 0.2 ^a^
DT10	11.4 ± 0.1 ^a^	10.8 ± 0.1 ^b^	11.7 ± 0.1 ^a^
DT11	12.5 ± 0.1 ^ab^	12.3 ± 0.1 ^a^	12.9 ± 0.1 ^b^
DT12	13.5 ± 0.1 ^a^	13.3 ± 0.1 ^ab^	13.0 ± 0.1 ^b^
DT13	8.8 ± 0.1 ^a^	8.9 ± 0.1 ^a^	8.9 ± 0.1 ^a^
DT14	9.3 ± 0.1 ^a^	9.6 ± 0.1 ^b^	8.7 ± 0.1 ^c^
DT15	10.0 ± 0.1 ^a^	10.4 ± 0.1 ^b^	10.1 ± 0.1 ^ab^
DT16	10.6 ± 0.1 ^a^	11.0 ± 0.1 ^b^	10.1 ± 0.1 ^c^
DT17	11.5 ± 0.1 ^a^	11.8 ± 0.1 ^b^	11.7 ± 0.1 ^ab^
DT18	3.4 ± 0.0 ^a^	3.3 ± 0.0 ^b^	3.5 ± 0.0 ^a^
DT19	3.6 ± 0.1 ^a^	3.2 ± 0.1 ^b^	4.0 ± 0.1 ^c^
DT20	3.2 ± 0.1 ^a^	3.1 ± 0.1 ^a^	3.5 ± 0.1 ^b^
DT21	5.0 ± 0.1 ^a^	4.8 ± 0.1 ^a^	5.3 ± 0.1 ^b^
DT22	4.8 ± 0.1 ^a^	4.6 ± 0.0 ^b^	4.9 ± 0.1 ^a^
DT23	3.6 ± 0.0 ^a^	3.7 ± 0.0 ^b^	3.6 ± 0.0 ^a^
DT24	2.5 ± 0.0 ^a^	2.5 ± 0.0 ^a^	2.4 ± 0.0 ^a^
DT25	2.5 ± 0.0 ^a^	2.6 ± 0.0 ^a^	2.5 ± 0.0 ^a^

**Table 4:** Overall and pairwise PERMANOVA comparisons for 25 body morphometrics distances among the three sampling sites in southeastern and southern Brazil of *Pomatomus saltatrix*. Abbreviations: DF, degrees of freedom; SS, sum of squares; R^2^, proportion of variation explained; F, pseudo-F statistic; *p*, permutation-based *p*-value; *p*-Adjusted, Bonferroni-adjusted *p*-values; RJ, Rio de Janeiro; SP, São Paulo; SC, Santa Catarina.

**Overall PERMANOVA**						
**Source**	**DF**	**SS**	**R^2^**	**F**	***p***	
**Site**	2	96.880	0.142	8.797	<0.001	
**Residual**	106	583.695	0.858			
**Total**	108	680.574	1.000			
**Pairwise PERMANOVA**						
**Groups**	**DF**	**SS**	**R^2^**	**F**	***p***	***p*** **-Adjusted**
**RJ–SP**	1	42.311	0.086	6.763	<0.001	<0.001
**RJ–SC**	1	33.874	0.074	6.337	<0.001	0.001
**SP–SC**	1	74.365	0.202	15.394	<0.001	<0.001

**Table 5:** Jackknifed cross-validation reclassification matrix obtained from Flexible Discriminant Analysis (FDA) based on body truss network measurements of *Pomatomus saltatrix* individuals collected from Rio de Janeiro (RJ), São Paulo (SP) and Santa Catarina (SC).

	Predicted Site				
Original Site	Leave-One-Out Cross Validation			Re-Classification	
	RJ	SP	SC	% of Correct Re-Allocation	% of Overall Re-Allocation
**RJ**	41	3	2	89.1	
**SP**	3	31	1	88.6	87.2
**SC**	4	1	23	82.1	

## Data Availability

The data presented in this study are available from the corresponding author upon reasonable request.

## Abbreviations

The following abbreviations are used in this manuscript:

ANOVA	Analysis of Variance
DT	Transformed Morphometric Distance
ESU	Evolutionarily Significant Unit
FDA	Flexible Discriminant Analysis
L50	Total Length at 50% Sexual Maturity
MOTU	Molecular Operational Taxonomic Unit
PERMANOVA	Permutational Multivariate Analysis of Variance
R^2^	Coefficient of Determination
RJ	Rio de Janeiro
SC	Santa Catarina
SL	Standard Length
SP	São Paulo
TL	Total Length
